# Lipid Profile in Multiple Sclerosis: Functional Capacity and Therapeutic Potential of Its Regulation after Intervention with Epigallocatechin Gallate and Coconut Oil

**DOI:** 10.3390/foods12203730

**Published:** 2023-10-11

**Authors:** Jose Enrique de la Rubia Ortí, Jose Luis Platero Armero, María Cuerda-Ballester, Claudia Emmanuela Sanchis-Sanchis, Esther Navarro-Illana, Jose María Lajara-Romance, María Benlloch, Jose Joaquín Ceron, Asta Tvarijonaviciute, Belén Proaño

**Affiliations:** 1 Department of Nursing, Catholic University of Valencia San Vicente Mártir, 46001 Valencia, Spain; joseenrique.delarubi@ucv.es (J.E.d.l.R.O.); joseluis.platero@mail.ucv.es (J.L.P.A.); ce.sanchis@ucv.es (C.E.S.-S.); esther.navarro@ucv.es (E.N.-I.); beprool@mail.ucv.es (B.P.); 2 Doctoral Degree School, Health Sciences, Catholic University of Valencia San Vicente Mártir, 46001 Valencia, Spain; maria.cuerda@ucv.es; 3 Multimedia Department, Catholic University of Valencia San Vicente Mártir, 46001 Valencia, Spain; jlajara@ucv.es; 4 Interdisciplinary Laboratory of Clinical Analysis, Campus of Excellence Mare Nostrum, University of Murcia, 30100 Murcia, Spain; jjceron@um.es (J.J.C.); asta@um.es (A.T.)

**Keywords:** multiple sclerosis, lipid profile, ketone bodies, coconut oil, EGCG

## Abstract

Background: Multiple sclerosis (MS) patients present dyslipidemia and functional disability. Epigallocatechin gallate (EGCG) and coconut oil have been shown to be effective against dyslipidemia. Objective: To analyze the relationship between lipid profiles, fat consumption, and functional disability in patients with MS after administering EGCG and coconut oil. Methods: A four-month pilot study was conducted on 45 MS patients, divided into an intervention group (IG) and a control group (CG). The IG received 800 mg of EGCG and 60 mL of coconut oil. Lipid profiles were measured before and after the intervention, along with other data such as dietary habits, inflammatory markers, and functional capacity. Results: Dyslipidemia did not correlate with the patients’ fat consumption. After the intervention, triglycerides (TG) levels were lower in IG compared to CG. This decrease was positively correlated with an improvement in functional disability (determined by the Expanded Disability Status Scale (EDSS)) and negatively with high-density cholesterol (HDL) and apolipoprotein A1. Significant and positive correlations were observed between EDSS and C-reactive protein (CRP) in the IG. These changes in the IG could be related to body fat decrease, whose percentage shows a positive correlation with CRP and TG levels, and a negative correlation with HDL levels. Conclusions: Patients with MS present a certain type of dyslipemia not associated with their nutritional habits. The administration of EGCG and coconut oil seems to decrease blood TG levels, which could explain the functional improvements.

## 1. Introduction

Multiple sclerosis (MS) is a chronic, neurodegenerative, and inflammatory disease characterized by a loss of myelin in the neuronal axons [1]. Clinically, there is a loss of muscle mass [2] and progressive functional deterioration [3], while from a pathophysiological point of view, there is a high oxidative stress [4] and mitochondrial dysfunction [5]. In addition, poor nutritional habits have shown a negative impact on the onset of the disease [6]. In this regard, it is widely accepted that peripheral levels of total cholesterol (TC), low-density lipoproteins (LDL), and triglycerides (TG) increase because of these poor habits, which are risk factors for the development of atherosclerosis, cardiovascular diseases, and metabolic disorders such as hypertension, insulin resistance, or dyslipidemia [7]. Alterations in cholesterol metabolism have also been linked to the pathophysiology of the disease [8], highlighting its possible role at the central level. The study of alterations in the lipid profile and its oxidation products has demonstrated diagnostic and therapeutic potential in neurodegenerative diseases [7,9]. In the specific case of MS, it is accepted that 31% of MS patients in Spain present dyslipidemia [10]. However, there is no consensus on its origin. Some studies indicate higher cholesterol levels in MS patients compared to healthy individuals, while others show a lower frequency of hypercholesterolemia compared to the general population [11] In any case, there is evidence of the relationship between dyslipidemia and disease progression and an association is seen between higher degrees of functional disability (according to the Expanded Disability Status Scale (EDSS)) and higher levels of TC, LDL and very-low-density lipoproteins (VLDL) [12,13,14]. Elevated levels of high-density lipoproteins (HDL) have been described in patients with MS and they present structural changes that lead to an alteration in its anti-inflammatory function [15] related to the characteristic fatigue of the disease [16]. It has also been seen that apolipoprotein A-1 (Apo A1) levels are lower in patients with MS compared to those in healthy individuals; and in experimental allergic encephalomyelitis-induced Apo AI-deficient mice, there was increased T-cell penetration into the CSF that led to an increase in demyelination [17]. Finally, it should be noted that the relationship between TG levels is especially relevant, both with functional disability [13] and with relapses in the disease [13]. This same relationship has been seen with VLDL levels, its main transporting lipoprotein [14]. In the central nervous system (CNS), TG may be involved in cognitive function due to their regulatory role in the pathways associated to appetite and their ability to cross the blood–brain barrier (BBB) [18]. It has been found that cerebrospinal fluid presents 0.6% of the TG levels, and, in animal models, the reduction of 46% of TG in blood by the action of the anesthetic gemfibrozil improved learning and memory [16].

There is currently no cure for MS and different nutrients or antioxidant molecules stand out as possible alternatives that may reverse or reduce the progression of the disease. Among these, the polyphenolic antioxidant epigallocatechin gallate (EGCG) has achieved multiple benefits in neurodegenerative diseases models such as Alzheimer’s and Parkinson’s [19,20], and specifically in MS, it is presented as an effective and safe candidate [21]. In addition, among the nutrients capable of improving the altered mitochondrial activity in MS, ketone bodies stand out, with coconut oil being an excellent precursor of these metabolites due to its high content of medium-chain fatty acids (MCFAs) [22]. These ketone bodies, and especially beta-hydroxybutyrate (βHB), have great benefits in neurodegenerative diseases [23], particularly in the CNS of the MS murine model, where behavioral, molecular, and morphological improvements were seen [24]. It is due to all of these reasons that the combined treatment of EGCG and increased βHB in the blood could promote lipid modulation activity, both through the action of the antioxidant [25,26,27] thanks to its effect on the expression of miRNAs in the liver [28], and through the increase in ketone bodies linked to improvements in hyperlipidemia [29,30]. In addition to this activity, we must add the anthropometric improvement promoted by EGCG and ketone bodies, by increasing lean mass and decreasing body fat [31,32,33,34], closely related to lipid metabolism.

Therefore, considering these discrepancies in serum lipoprotein levels, and their possible influence on the progressive functional disability of the disease, the objective of the present study was to describe the lipid profile of patients with MS and its relationship with nutritional habits, as well as the possible improvement of functional capacity and the involvement of different lipoproteins in these changes, after EGCG and coconut oil intake.

## 2. Materials and Methods

**Study design and intervention**

A pilot, descriptive, quantitative, and experimental study was conducted. The clinical trial ID was NCT03740295. The population sample was obtained from the main state-wide MS associations who were previously informed about the study. The selection criteria and consort diagram flow for the experimental sample, consisting of 51 patients with MS randomly distributed into a control group (CG) and an intervention group (IG), is described in the study by Benlloch et al. [35], conducted in our lab; the participants considered for the present study were the same, except for one patient that was excluded from the analysis because their TG were 4SD higher than the mean (Figure S1). The IG was administered 800 mg of EGCG and 60 mL of coconut oil daily for 4 months, as well as an isocaloric diet, which was also followed by the CG. In this previous study, the selected patients’ functionality (EDSS scale), metabolic and inflammation markers in serum (C-reactive protein (CRP), paraoxonase 1 (PON1), and βHB), and anthropometric parameters (body mass index (BMI), fat mass, and muscle mass) were already determined.

**Serum extraction procedure and lipid profiling**

Before and after the intervention, fasting blood samples were obtained from all participants. These samples were centrifuged to separate serum from plasma, and the TC, LDL, HDL, Apo A1, and TG levels were measured from the plasma. All the analytes were calculated by commercially available assays (Beckman Coulter) in an automated biochemistry analyzer (Olympus A400, Beckman Olympus AU400 Chemistry Analyzer, Beckman Coulter, Brea, CA, USA).

**Nutritional analysis**

**Nutritional Dietary Anamnesis**

Each patient in the population sample underwent an anamnesis to obtain information regarding certain individual aspects such as personal and family history, usual medical treatments, comorbidities, food allergies or intolerances, and physical activity habits and the Food Frequency Questionnaire (FFQ) was employed [36]. Likewise, a food diary, was used as a supporting tool. Each patient had to record their daily intake, both solids and liquids, for 7 days. This time period allowed us to decrease the bias associated with the choice of a single day of the week, and in turn, obtain sufficient information about the usual diet of each patient [37]. The patient had to record the type of food in the food diary, as well as the ingredients consumed. In addition, they had to specify the amounts, how they were measured, and ingredients in each intake. To facilitate this task, information was previously provided regarding the most common way of weighting food portions at home [38].

**Eating habits and dietary analysis**

Likewise, an assessment of the patients’ diet was carried out through the nutritional-dietary software “EasyDiet^®^—Consultation Management Program” EasyDiet^®^ (www.easydiet.es) [39]. The data that patients provided in their dietary records were entered into the program to obtain their nutritional profile. Values considered for analysis in the present work were total lipids (g), cholesterol (mg), and saturated fats.

**Statistical analysis**

Lipid profile values were available for the same 46 patients in which inflammatory markers were previously analyzed [35]. Only one patient was excluded from the analysis because of TG levels above 4SD from the mean (z-score = 4.31), resulting in a sample of 45 MS patients. Mean comparisons for control and intervention groups were performed for age, time since diagnosis, and lipid values. For regression and correlation analysis, these data were combined with functional, anthropometric, dietary, and inflammatory data of the same population.

Statistical analysis was performed with the program SPSS v.23 (IBM Corporation, Armonk, NY, USA). Categorical variables were reported as proportions and compared with chi-square test or Fisher’s exact test, when at least 25% of cells had expected counts less than 5. Continuous variables were reported as mean and standard deviation and normality was assessed by Shapiro–Wilk test. Comparison of means between groups was made with Student’s *t* test for independent samples or the Mann–Whitney U test, for normal or non-normal distributed data, respectively. Comparisons of means within groups to determine effect of the treatment were made with Student’s *t* test for dependent samples or the Wilcoxon test, for normal or non-normal distributed data, respectively.

In addition to the mean comparisons of lipid profile values, linear regression models were constructed for a better understanding of the associations of the lipid profile with functionality and inflammatory process prior the intervention. No more than 7 predictor variables were used in each model, the combination of variables was chosen to avoid collinearity, with a variance inflation factor (VIF) < 2. To assume the independence of errors, values between 1.5 and 2.5 in the Durbin–Watson test were accepted. For each functional (EDSS, ten-meter walk test (10MWT), and two-minute walk test (2MWT)), anthropometric (muscle mass and fat mass), or dietary (total lipids, saturated fats, and cholesterol) parameter lipid profile molecules were used as predictor variables, together with possible confounders such as: sex, age, BMI, and EDSS. For the EDSS model, only sex, age, and BMI were placed as confounders. For anthropometric variables (fat mass and muscle mass), BMI was excluded from the analysis. Finally, associations after the intervention were assessed by partial Pearson correlations controlling for age and sex in both the control and intervention groups. The level of significance in all analyses was α < 0.05.

**Ethical Considerations**

The study was developed in accordance with the Declaration of Helsinki [40], once the protocol was approved by the Human Research Ethics Committee of the Experimental Research Ethics Committee of the University of Valencia (procedure number H1512345043343). The study protocol was approved by the Ethics Committee of the Universidad de Valencia.

## 3. Results

Anthropometric and lipid profile values analyzed by sex are shown in Table 1. In this population sample, there were 15 men and 30 women, with an average age of 47 years, a time since diagnosis of 13 years, and a BMI of 25 kg/m^2^. The general levels of TC, TG, and LDL in the patients of the sample were 217 mg/dL, 107 mg/dL, and 132 mg/dL, respectively. The HDL and Apo A1 levels were 83 mg/dL and 175 mg/dL, respectively. No statistically significant differences were found between men and women in any of the parameters described, except for Apo A1 concentrations, which were 13 mg/dL higher in women. No significant differences were found in the proportions of MS type between men and women, with a higher proportion of relapsing–remitting MS for both sexes.

### 3.1. Lipid Profile in Relation to Anthropometric Parameters

Regression analysis for lean mass and muscle mass with lipid profile parameters showed a significant effect only for LDL levels on both parameters; all coefficients and significance values can be seen in Table 2. In the relationship between fat mass and TC, the model was statistically significant (*p* < 0.001) due to the effect of BMI. However, when each type of lipoprotein was explored, LDL showed a significant positive association (β = 0.319, *p* = 0.003), indicating that high levels of this molecule are associated with a higher percentage of fat mass, as well as a significant effect of sex and BMI, with a total variability of 76.4% explained in this model (F = 15.66, *p* < 0.001). For muscle mass, a relationship with LDL was also observed, which in this case is negative (β = −0.48, *p* = 0.001), indicating that for a high percentage of muscle mass there are lower levels of LDL, with a significant effect of sex and level of disability, adding up to a total of 52.8% of the variability explained in this model (F = 5.43, *p* < 0.001). In addition, muscle mass showed a significant negative relationship with TC levels (β = −0.303, *p* = 0.025), with a significant effect of sex (F = 5.07, *p* = 0.001).

### 3.2. Lipid Profile in Relation to Functionality

The EDSS did not show any association with the lipid profile (Table S1). However, significant negative relationships were found between TC, LDL, and HDL with gait (Table 2). It was observed that patients with higher TC and HDL levels were slower, obtaining lower values in the 10MWT, and covered fewer meters in the 2MWT (β = −0.359). Additionally, patients with higher HDL levels were also slower (β = −0.288).

### 3.3. Nutritional Habits of the Study Population

**Figure 1:**
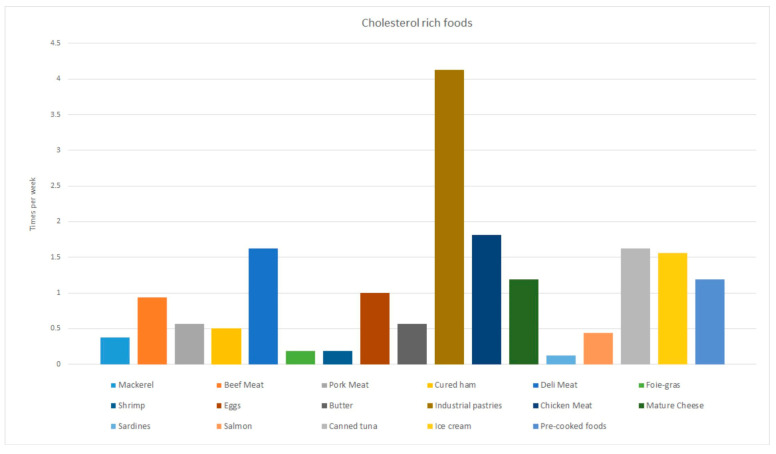
Weekly consumption of the main cholesterol rich foods by patients with multiple sclerosis (MS).

After analyzing the dietary records of the 45 patients with MS who participated in the current study, it was seen that they consumed a high amount of foods rich in fats, among which the high consumption of simple sugars (industrial bakery, chocolate, soft drinks, juices, dairy desserts), processed meats, and snacks stands out, as shown in Figure 1. Patients consumed industrial desserts such as cookies, muffins, and pre-packaged cakes, among others, 4 times a week on average. They also consumed ice cream 1.5 times a week on average, as well as canned tuna and deli meat. A consumption of up to 1.2 times a week of pre-cooked foods was also observed.

### 3.4. Relationship between Nutritional Habits and Lipid Profile

Since the intake of foods high in fat is a source of cholesterol and saturated fats, regression analysis was performed for the lipid profile and intake of total lipids, saturated fats, and cholesterol. No relationship was found between these parameters, nor was there a significant effect of any of the confounding variables (age, sex, or EDSS). The coefficients and statistical significance can be seen in Table 3.

### 3.5. Effect of the Intervention

Participants in the CG (*n* = 20) and IG (*n* = 25) presented the characteristics and initial levels indicated in Table 4. No statistically significant differences were found in the type of MS, age, or in any initial lipid profile, although there was a higher proportion of women in the IG (80% vs. 20%, *p* = 0.034).

#### 3.5.1. Changes in Blood Lipoprotein Levels

**Figure 2:**
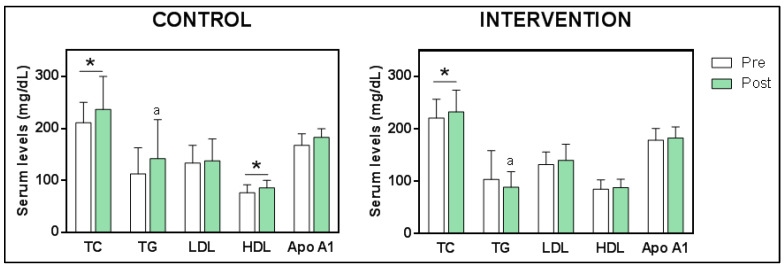
Lipid profile before and after intervention in control and intervention groups. * *p* < 0.05 for within comparison, a: *p* < 0.05 for between comparison. Abbreviations: TC: total cholesterol; TG: triglycerides; LDL: low-density lipoprotein; HDL: high-density lipoprotein; Apo A1: Apolipoprotein A-1.

Lipid profile parameters showed some differences after treatment (Figure 2). TC levels increased significantly in both groups, from 210.8 ± 39.39 mg/dL to 236.4 ± 63.2 mg/dL (t _16_ = −2.790; *p* = 0.013) in the CG, and from 220.9 ± 35.96 mg/dL to 232.5 ± 41.89 mg/dL (z _24_ = −2.085; *p* = 0.037) in the IG. HDL increased only in the CG, from 76.6 ± 15.2 mg/dL to 86 ± 14.2 mg/dL (z _16_ = −2.533; *p* = 0.011); in the IG, HDL levels remained constant around with 84.8 ± 18.1 mg/dL and 87.9 ± 16.2 mg/dL in the pre and post measurements, respectively. TG levels did not show significant changes in any of the groups (in the CG from 112.8 ± 50 mg/dL to 142 ± 75 mg/dL, and in the IG, from 103.6 ± 55 mg/dL to 88.7 ± 30 mg/dL (z _20_ = −1.399; *p* = 0.162)). However, when lipid parameters after treatment were analyzed by two independent sample means, only TG levels showed significant differences, with lower TG levels in the IG than in CG (z _22_ = −2.201, *p* = 0.028). The other parameters, LDL and Apo A1, did not show significant differences in any of the groups.

#### 3.5.2. Relationships between Lipid Profile, Functionality, Anthropometry and Inflammation Markers after Intervention

**Figure 3:**
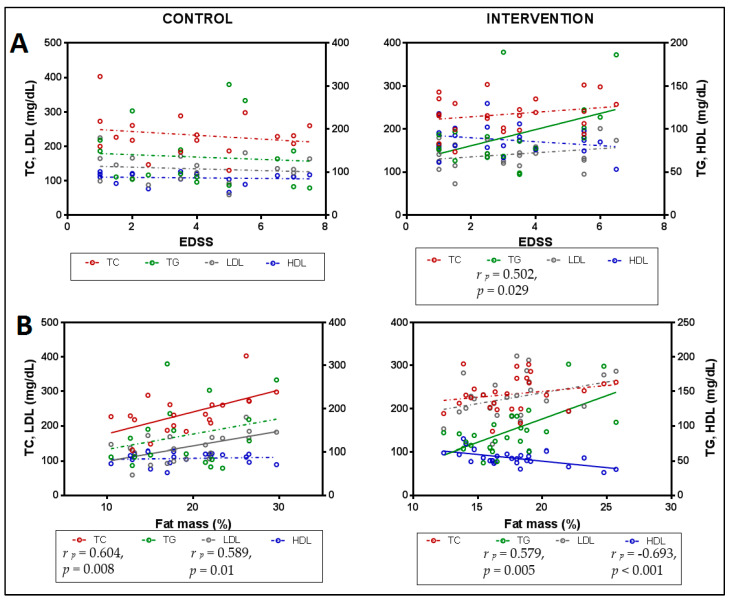
Correlation analysis for lipid profile with: (**A**). functionality and (**B**). fat mass after intervention in both the control and intervention groups. Partial correlation coefficients (r _p_) and *p*-values are shown when the association was statistically significant (continuous line). Partial correlations were made controlling for age and sex, dashed lines represent the trend of non-statistically significant correlations. Abbreviations: TC: total cholesterol; TG: triglycerides; LDL: low-density lipoprotein; HDL: high-density lipoprotein; EDSS: Expanded Disability Status Scale.

The lipid profile showed relationships with functionality parameters only in the IG (Figure 2). EDSS showed a statistically significant positive association with TG, indicating that lower levels of TG were associated with lower levels of disability. On the other hand, the level of disability was not related to any molecule of the lipid profile in the CG (Figure 3A).

Similarly, body fat showed different associations with the lipid profile depending on the group (Figure 3B): in the CG, significant associations were observed with the levels of TC and LDL, indicating that higher levels of these molecules were associated with a higher percentage of body fat, while in the IG, a positive relationship was observed with levels of TG and a negative association with levels of HDL, indicating that lower body fat levels were associated with lower TG levels and higher HDL levels.

**Figure 4:**
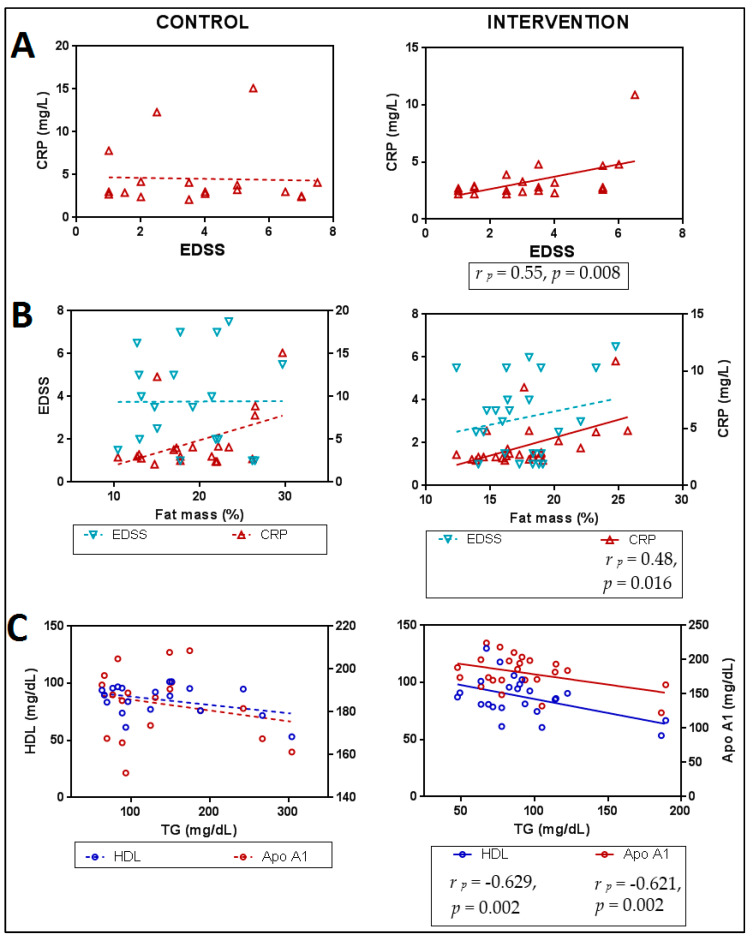
Correlation analysis for metabolic and inflammatory markers with: (**A**). functional, (**B**). anthropometric and (**C**). lipid parameters after intervention in both, control and intervention groups. Partial correlation coefficients (r _p_) and *p*-value are shown when the association was statistically significant (continuous line). Partial correlations were made controlling for age and sex, dashed lines represent the trend of non-statistically significant correlations. Abbreviations: TC: total cholesterol; TG: triglycerides; LDL: low-density lipoprotein; HDL: high-density lipoprotein; CRP: C-reactive protein; EDSS: Expanded Disability Status Scale; Apo A1: apolipoprotein A-1.

Finally, only in the IG were significant positive associations found between the levels of CRP and both disability (EDSS) (Figure 4A) and the percentage of body fat (Figure 4B), indicating that lower levels of disability and body fat were associated with lower levels of CRP. The degree of disability was not associated with body fat in any group (Figure 4B). It should be noted that TG levels were negatively and significantly associated with HDL levels (r p = −0.629, *p* = 0.002) and Apo A1 (r p = −0.62, *p* = 0.002) only in the IG (Figure 4C), indicating that the decrease in TG was associated with increases in the levels of both molecules.

## 4. Discussion

Lipid control is common in clinical practice as it is a risk factor for cardiovascular diseases, although it is also beginning to be linked with neurodegeneration [41,42]. Special attention is given to serum TC and LDL, whose maximum recommended values in a healthy European population are ~190 mg/dL and ~115 mg/dL, respectively. The limit for fasting TG is ~150 mg/dL. As for HDL, values above 40 mg/dL in men and 45 mg/dL in women are recommended, with values between 119 and 240 mg/dL being accepted as normal for Apo A1 [43].

In our sample, we were able to determine a dyslipidemia characterized by high levels of TC and LDL: 217 mg/dL and 132 mg/dL, respectively. The values of Apo A1 and TG were within recommended limits: 175 mg/dL and around 100 mg/dL, respectively. Another aspect to highlight is the high levels of HDL, around 80 mg/dL. Metabolic alterations, including dyslipidemia, have already been suggested in this disease. Recent published data show that MS patients with a similar age range to the present sample (between 40 and 55 years) also had at-the-limit or above recommended TC and LDL levels [10,13,44,45]. The altered regulation of lipid metabolism in MS may be partly due to nutritional habits, which negatively influences the disease as already seen in a previous publication for this same group of patients [46]. The consumption of ultra-processed foods, such as deli meats, industrial pasta, pastries, and ready-to-eat meals, increases TG, LDL, and TC levels, since these products are rich in saturated and hydrogenated fats. Using these products excessively can raise the risk of developing metabolic syndrome by elevating the levels of this lipoprotein in serum, as seen in a study by Canhada SL, et al., 2023 [47]. However, in our population, the dietary pattern of these foods related to the lipid profile was not so unbalanced; thus, lipid values were not associated with the weekly intake of total lipids, saturated fats, or cholesterol (Table 3), which have been linked to an increase in TG and TC through exogenous synthesis in healthy populations. This suggests that dyslipidemia in patients with MS is part of the pathophysiological mechanism of the disease. Precisely, in other studies, dyslipidemia in MS has been linked to both a higher degree of functional disability [48] and a worse prognosis. In this context, an association between lipid and lipoprotein values with EDSS has not been seen, but there is a correlation with walking ability, which directly influences the disability of these patients [49]. Along these lines, our team already reported an improvement in the functional capacity of the patients with MS in this sample after the described intervention [50]. This is why, in the current study, we aim to analyze the possible link between this improvement and treatment through the regulation of the lipid profile, because coconut oil and EGCG have already demonstrated this modulating activity in humans, animal models, and cellular models [25,26,27,29,30].

After the 4-month-intervention, it should be noted that the only significant difference between groups was due to a decrease in TG levels seen in the IG (Figure 2). This change could explain the improvement in functional capacity, as a significant positive correlation was found between TG levels and scores on the EDSS disability scale only in this group (Figure 3A). Patients who showed the greatest decrease in TG values were those who demonstrated the greatest functional improvement, confirming the relationship between TG levels and functional capacity previously suggested [13]. Furthermore, this group presented significant and negative correlations between TG levels with HDL and Apo A1 (Figure 4C), clearly supporting the role of EGCG in regulating the lipid balance previously seen by Gutiérrez-Salmeán et al., 2016, after treatments with catechins in patients with hypertriglyceridemia [51].

It should be noted that no changes in HDL levels were found in the IG, but significant increases were observed in the CG, which could be due to the effect of the healthy baseline diet received for 4 months (starting from slightly lower HDL levels than those found in the IG), coinciding with what was seen by Fellows et al., 2019, where HDL levels also increased after dietary intervention in a population with progressive MS [44]. However, previous studies showed that a decrease in ultra-processed food intake only stabilizes levels of TC, LDL, and TG [52]. Thus, the elevated levels of HDL found in the sample, while raising a question, may provide a crucial factor in elucidating dyslipidemia and its role in the pathogenesis of MS. In this sense, it is important to highlight that, for patients not subjected to any type of treatment, high levels of HDL are shown to be dysfunctional in the disease, as they do not perform their anti-inflammatory activity [15]. For this reason, the fact that the CG significantly increases TC and HDL could be rather due to a progression of the disease at the inflammatory level. On the other hand, the IG that received the treatment did not show significant changes in HDL levels, although it did in TC, suggesting an alteration in the lipid balance that could be beneficial for patients. In this regard, the results of the present analysis lead us to think that a change in the paradigm and composition of HDL molecules occurs only in the IG. This change would mean that, although HDL levels remain around 80 mg/dL after treatment, their anti-inflammatory activity is restored, a change that could be partly related to the significant increase in PON1 enzyme levels, previously reported only for the IG [35]. Thus, in an environment with higher levels of PON1, HDL could become functional again, acting as a transporter of PON1, characterized by its powerful anti-inflammatory and antioxidant activity [53,54,55]. In addition to PON1, the aforementioned Apo A1 also appears to be important. This is because higher levels of Apo A1, not just HDL, are linked to less damage to the BBB in patients with MS [56], and it is interesting to note that, after treatment in the IG, not only are the patients who lower their TG levels the ones with the highest HDL levels, but they also show the highest levels of Apo A1 (Figure 4C). According to the existing literature, HDL levels, specifically through the action of Apo A1, are related to myelin improvement [57], as well as an increase in oligodendrogenesis, axonal regeneration [58], and reduced extravasation of immune cells in the CNS, which implies a decrease in inflammation at the central level [59]. All these mechanisms involving HDL could, in turn, help to explain the improvement in walking capacity already evidenced in our laboratory by the IG group; specifically, the increase in walking speed (10MWT), which is the variable that improves only in this group [60] and is negatively associated with HDL values in our population sample before the intervention (Table S1).

To understand how our treatment achieves a paradigm shift in the lipid profile, possibly based on the new role of HDL and also related to the decrease in TG, we need to analyze the activity of both EGCG and the ketone bodies promoted by the intake of coconut oil. Regarding EGCG, it stands out that it upregulated autophagic lipolysis in adipocytes [61], which could explain the previously reported significant direct correlation of the decrease in fat mass [33] with the levels of inflammation determined by CRP (Figure 4B). Accordingly, based on recent findings by Doğan et al. which showed that people with less body fat have higher levels of PON1 than obese people [62], the importance of the interaction between body fat and the regulation of lipid metabolism would be confirmed, leading to a restoration of the anti-inflammatory role of HDL, which correlates negatively with the percentage of fat after treatment (Figure 3B), as well as for TG (Figure 4C). There is also evidence that EGCG decreases blood TG levels [63], as well as TG content in the liver in an animal model after administering a high-fat diet and, subsequently, EGCG and a lycopene lycophylic [64].

This is consistent with our results, where no changes in LDL concentrations occur after the intervention. Moreover, this lipolytic activity promotes a decrease in deposited TG and the synthesis of free fatty acids, which cannot only increase the production of ketone bodies in the liver but also promote the remyelination of neurons after crossing the BBB [65]. It is also worth remembering that EGCG itself is capable of crossing the BBB [66] allowing it to accumulate centrally. The crossing of the BBB can be highly beneficial for patients with MS due to the demonstrated neurogenic activity of EGCG [67], which is combined with its high anti-inflammatory action [68,69]. Regarding the activity of increasing blood ketone bodies promoted by hepatic β-oxidation after ingestion of coconut oil, it should be noted that these metabolites, upon binding to GPR10A receptors (activated by niacin used in the treatment of dyslipidemia), increase the levels of HDL in the blood, which would presumably produce the described benefits [70]. The role of ketone bodies, especially βHB, in the central nervous system is also important, as it has been shown that they restore neuronal myelin and reduce levels of reactive oxygen species (ROS) and inflammation [71]. Therefore, inflammation is reduced through both pathways (EGCG and increased ketone bodies), which is associated with improved myelin and recently linked to an increase in muscle mass [72].

At this point, it is interesting to note that the lipid profile influences inflammation and vice versa. Specifically, on the one hand, lipoproteins rich in TG and TG themselves are associated with an increase in the production of proinflammatory cytokines and the activation of monocytes [73] and, on the other hand, during an acute phase reaction, the levels of HDL-C and Apo A1 are reduced, and the proteins associated with HDL are altered, which affects the antioxidant and anti-inflammatory properties of HDL [74].

As brilliantly established by Cho et al., 2020 regarding neuromyelitis optica spectrum disorder, serum levels of TG show a positive association with EDSS scores, with changes in HDL levels depending on the disease phase [75]. Therefore, it seems plausible that our treatment can lead to functional improvements through the reduction in inflammation, resulting from changes in TG levels and their relationship with HDL. Furthermore, confirming that functional improvement is directly linked to the reduction in inflammation, it should be noted that only patients who received the treatment show a positive correlation between EDSS and serum CRP levels (Figure 3A). Thus, those who show the most functional improvement also have lower levels of CRP. There is no consensus to date about the use of this protein in disease diagnosis and progression [76], but changes have been seen during relapses compared to periods of remission [77] that imply functional worsening. Additionally, CRP is a good marker of the state of the vascular endothelium and elevated levels of CRP are not only associated with inflammation but also with BBB alteration and EDSS [78]. Thus, an improvement in the state of inflammation and the BBB would justify this same correlation seen between EDSS and CRP levels in the IG.

**Figure 5:**
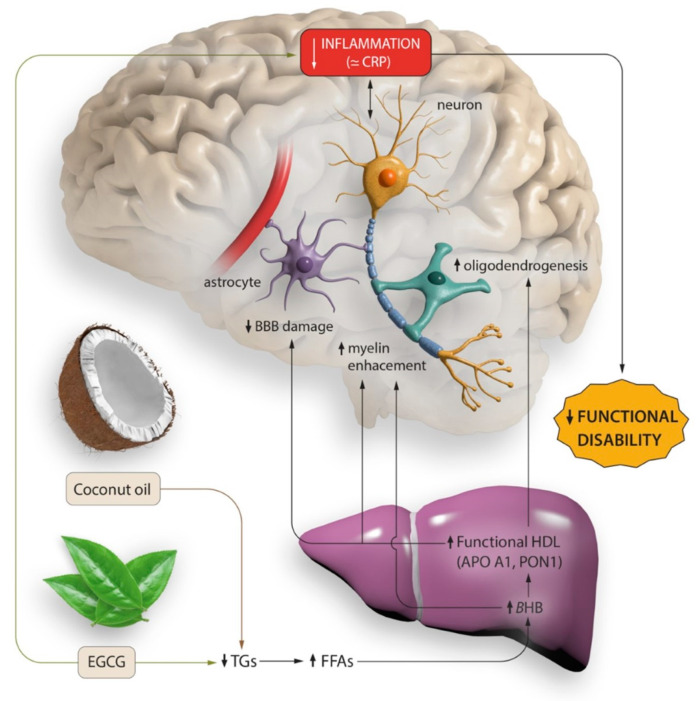
Possible effects on functional disability of lipid profile change after treatment with EGCG and coconut oil.

All of these hypotheses and results are shown in Figure 5.

### Limitations of the Study

Despite these findings, our study has limitations. Being a pilot study, the population sample is small and a more representative population is required. In addition, it would be interesting to explore the change in HDL composition, quantifying the different subtypes. Finally, a certain degree of inaccuracy cannot be ruled out in food diaries that are self-reported by the patient and the possibility of this bias should be considered. Therefore, it is necessary to further investigate lipid metabolism and its role in the progression and prognosis of MS.

## 5. Conclusions

As a conclusion, patients with MS show dyslipidemia characterized by elevated values of TC, LDL, and HDL, while TG and Apo A1 are within normal range. These lipid levels are not associated with nutritional habits. In addition, EGCG and coconut oil improve the functional capacity of patients with MS. This improvement could be due to the treatment’s impact on reducing blood TG levels; thus, TG in particular could be especially relevant as a prognostic biomarker of the disease.

## Figures and Tables

**Table 1:** Description of anthropometric, lipid, and clinical characteristics in the subsample of multiple sclerosis (MS) patients.

	Overall (*n* = 45)	Male (*n* = 15)	Female (*n* = 30)	
	M ± SD	M ± SD	M ± SD	*p*-Value
Age (y)	47 ± 12.6	46.9 ± 13.4	47 ± 12.5	0.979 ^t^
Time since diagnosis (y)	13.1 ± 9.6	15.5 ± 9.1	11.9 ± 9.7	0.122 ^u^
BMI (kg/m^2^)	25.6 ± 5.8	25.2 ± 7.1	25.7 ± 5.3	0.613 ^u^
TC (mg/dL)	217.4 ± 37.6	216.5 ± 44.2	217.9 ± 34.3	0.900 ^t^
TG (mg/dL)	107.1 ± 50.2	104.6 ± 38.6	108.5 ± 55.8	0.911 ^u^
LDL (mg/dL)	132 ± 27.7	134 ± 34.6	130.9 ± 23.6	0.725 ^t^
HDL (mg/dL)	83.2 ± 19.2	79 ± 22.1	85.6 ± 17.4	0.059 ^u^
Apo A1 (mg/dL)	175.6 ± 23.8	167.8 ± 25	179.9 ± 22.4	0.037 ^u^
**MS type**	**N (%)**	***n* (%)**	***n* (%)**	***p*-value**
Relapsing–Remitting	34 (75.6)	9 (60)	25 (83.3)	0.09 ^f^
Secondary-Progressive	11 (24.4)	6 (40)	5 (16.7)	

M: mean; SD: standard deviation; BMI: body mass index; TC: total cholesterol; TG: triglycerides; LDL: low-density lipoprotein; HDL: high-density lipoprotein; Apo A1: apolipoprotein A-1; MS: multiple sclerosis; M: mean; SD: standard deviation; ^t^: Student’s *t* test for independent samples; ^u^: Mann–Whitney U test; ^f^: Fisher’s exact test. Significant *p*-values are in bold.

**Table 2:** Linear regression model for anthropometric and functional parameters as outcome variables, with lipid parameters as independent variables before the intervention.

	Regression Coefficients: Fat Mass (%)					Regression Coefficients: Muscle Mass (%)				
	B	SE B	β	t	*p*-Value	B	SE B	β	t	*p*-Value
TC	0.017	0.010	0.164	1.69	0.098	−0.027	0.012	−0.303	−2.32	**0.025**
	R^2^ = 0.674; F_model_ = 15.32, *p* < 0.001; d					R^2^ = 0.407; F_model_ = 5.07, *p* = 0.001; b				
TG	−0.001	0.008	−0.010	−0.097	0.924	0.002	0.010	0.036	0.248	0.806
LDL	0.047	0.014	0.319	3.25	**0.003**	−0.061	0.017	−0.482	−3.48	**0.001**
HDL	−0.034	0.018	−0.162	−1.89	0.067	0.016	0.022	0.089	0.732	0.469
	R^2^ = 0.764; F_model_ = 15.66, *p* < 0.001; b, d					R^2^ = 0.528; F_model_ = 5.43, *p* < 0.001; b, c				
	**Regression coefficients: 10MWT**					**Regression coefficients: 2MWT**				
	**B**	**SE B**	**β**	**t**	***p*-value**	**B**	**SE B**	**β**	**t**	***p*-value**
TC	−0.004	0.002	−0.197	−1.55	0.132	−0.259	0.1	−0.267	−2.58	**0.014**
	R^2^ = 0.543; F model = 7.374, *p* < 0.001; a, b					R^2^ = 0.649; Fmodel = 13.279, ***p* < 0.001**; a, b				
TG	0.001	0.002	0.049	0.311	0.758	0.106	0.087	0.144	1.21	0.233
LDL	−0.001	0.004	−0.047	−0.315	0.755	−0.489	0.16	−0.359	−3.05	**0.004**
HDL	−0.01	0.004	−0.288	−2.22	**0.035**	−0.337	0.185	−0.176	−1.82	0.077
	R^2^ = 0.588; F _model_ = 5.731, ***p* < 0.001**; a, b					R^2^ = 0.71; F_model_ = 11.535, ***p* < 0.001**; a, b				
	**Regression coefficients: Berg**									
	**B**	**SE B**	**β**	***t***	***p*-value**					
TC	−0.086	0.036	−0.27	−2.366	**0.023**					
	R^2^ = 0.54; F model = 8.811, ***p* < 0.001**									
TG	0.047	0.032	0.2	1.463	0.153					
LDL	−0.164	0.056	−0.38	−2.929	**0.006**					
HDL	−0.019	0.071	−0.03	−0.263	0.794					
	R^2^ = 0.586; F_model_ = 6.87, ***p* < 0.001**; a, b									

Model controlling for age, gender, and the Expanded Disability Status Scale (EDSS). 2MWT: two-minute walk test; SE B: standard error for B coefficient; TC: total cholesterol; TG: triglycerides; LDL: low-density lipoprotein; HDL: high-density lipoprotein; a = effect of age; b = effect of sex; c = effect of EDSS; d = effect of body mass index (BMI). Significant *p*-values are in bold.

**Table 3:** Linear regression analysis for lipid profile molecules as outcome variables and dietary parameters as independent variables, previous the intervention.

	Regression Coefficients: Systemic LDL					Regression Coefficients: Systemic TC				
	B	SE B	β	*t*	*p*-Value	B	SE B	β	*t*	*p*-Value
Total lipids	0.555	0.358	0.334	1.549	0.138	0.718	0.498	0.323	1.441	0.166
	R^2^ = 0.25; F_model_ = 1.269; *p* = 0.318					R^2^ = 0.189; F_model_ = 0.886; *p* = 0.510				
Saturated fats	0.568	0.749	0.161	0.758	0.458	0.931	1.028	0.197	0.905	0.377
	R^2^ = 0.18; F_model_ = 0.836; *p* = 0.540					R^2^ = 0.138; F_model_ = 0.606; *p* = 0.696				
Cholesterol	0.067	0.072	0.218	0.938	0.360	0.127	0.097	0.307	1.303	0.208
	R^2^ = 0.193; F_model_ = 0.090; *p* = 0.496					R^2^ = 0.174; F_model_ = 0.802; *p* = 0.562				
	**Regression coefficients: systemic HDL**					**Regression coefficients: systemic TG**				
	**B**	**SE B**	**β**	**t**	***p*-value**	**B**	**SE B**	**β**	**t**	***p*-value**
Total lipids	−0.106	0.212	−0.119	−0.501	0.622	0.871	0.651	0.297	1.339	0.197
	R^2^ = 0.095; F_model_ = 0.4; *p* = 0.843					R^2^ = 0.246; F_model_ = 1.171; *p* = 0.361				
Saturated fats	−0.290	0.421	−0.153	−0.689	0.499	1.497	1.337	0.238	1.119	0.278
	R^2^ = 0.106; F_model_ = 0.449; *p* = 0.809					R^2^ = 0.225; F_model_ = 1.041; *p* = 0.424				
Cholesterol	0.008	0.041	0.048	0.194	0.848	0.163	0.125	0.292	1.297	0.211
	R^2^ = 0.085; F_model_ = 0.354; *p* = 0.873					R^2^ = 0.241; F_model_ = 1.145; *p* = 0.373				

Model controlling for age, gender and Expanded Disability Status Scale (EDSS). SE B: standard error for B coefficient; TC: total cholesterol; TG: triglycerides; LDL: low-density lipoprotein; HDL: high-density lipoprotein.

**Table 4:** Demographic and lipid profile information of the study group before treatment.

	Control Group	Intervention Group	
	*n* (%)	*n* (%)	*p*
MS type			
Relapsing-Remitting	16 (80)	18 (72)	0.729 ^f^
Secondary-Progressive	4 (20)	7 (28)	
**Sex**			
Men *n* (%)	10 (50)	5 (20)	0.034 ^c^
Women *n* (%)	10 (50)	20 (80)	
	**M ± SD**	**M ± SD**	***p***
Age (y)	48.9 ± 12.5	44 ± 11.5	0.176 ^t^
Time since diagnosis (y)	14.9 ± 9	11.7 ± 10	0.114 ^u^
BMI (kg/m^2^)	25.8 ± 6	25.9 ± 5.2	0.887 ^u^
TC (mg/dL)	210.8 ± 39.39	220.9 ± 35.96	0.873 ^u^
TG (mg/dL)	112.8 ± 50	103.6 ± 54.87	0.540 ^u^
LDL (mg/dL)	133.4 ± 34.1	131.9 ± 24.07	0.976 ^t^
HDL (mg/dL)	76.56 ± 15.22	84.81 ± 18.05	0.249 ^u^
Apo A1 (mg/dL)	167.6 ± 21.77	178.7 ± 22.54	0.126 ^u^

MS: multiple sclerosis; M: mean; SD: standard deviation; ^c^: chi-square test; ^f^: Fisher’s exact test; ^t^: parametric test; ^u^: non-parametric test; BMI: body mass index; TC: total cholesterol; TG: triglycerides; LDL: low-density lipoprotein; HDL: high-density lipoprotein; Apo A1: apolipoprotein A-1.

## Data Availability

The data used to support the findings of this study can be made available by the corresponding author upon request.

## Supplementary Materials

The following supporting information can be downloaded at: https://www.mdpi.com/article/10.3390/foods12203730/s1, Figure S1: consort diagram flow [79]; Table S1: EDSS association with the lipid profile.
