# Supplementary material for: Lipid Profile in Multiple Sclerosis: Functional Capacity and Therapeutic Potential of Its Regulation after Intervention with Epigallocatechin Gallate and Coconut Oil
Source: Foods. 2023 Oct 11;12(20):3730. doi: 10.3390/foods12203730 (PMC10606609; doi:10.3390/foods12203730)
Supplement: Supplementary file 1 [file foods-12-03730-s001.zip › supplementary Figure S1 CONSORT DIAGRAM FLOW.pdf]

Figure S1 CONSORT DIAGRAM FLOW

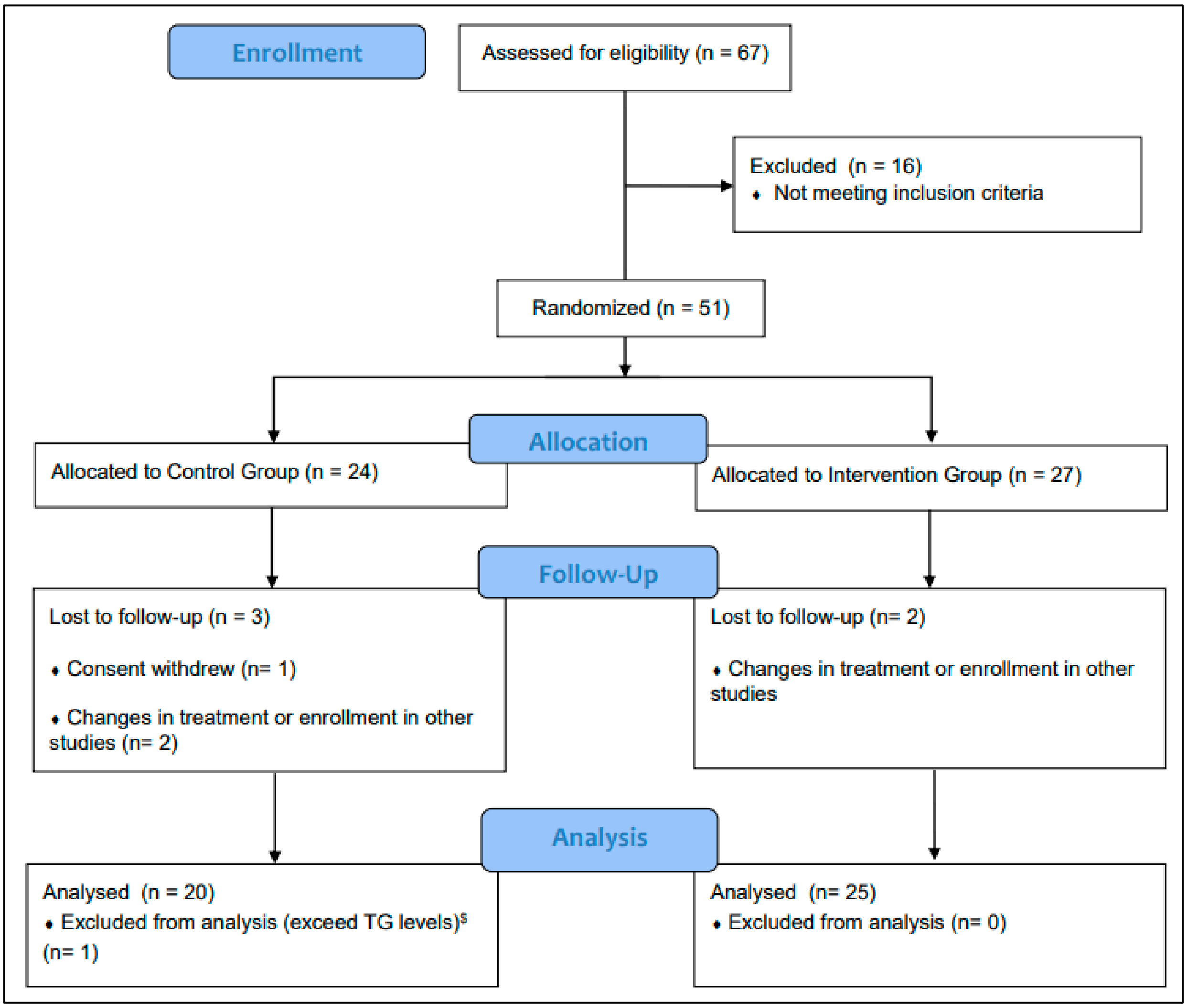

[$] TG levels were considered to exceed when serum levels (mg/dL) were above 4SD from the mean (z-score = 4.31). When TG levels exceed 400 mg/dl, it has been reported the need of a different equation to represent lipid profile molecules concentration (Sajja A, Park J, Sathiyakumar V, et al. Comparison of Methods to Estimate Low-Density Lipoprotein Cholesterol in Patients With High Triglyceride Levels. *JAMA Netw Open.* 2021;4(10):e2128817. doi:10.1001/jamanetworkopen.2021.28817).
