# Supplementary material for: Lipid Profile in Multiple Sclerosis: Functional Capacity and Therapeutic Potential of Its Regulation after Intervention with Epigallocatechin Gallate and Coconut Oil
Source: Foods. 2023 Oct 11;12(20):3730. doi: 10.3390/foods12203730 (PMC10606609; doi:10.3390/foods12203730)
Supplement: Supplementary file 1 [file foods-12-03730-s001.zip › Supplementary_Material Tabla S1.pdf]

Supplementary Material

Table S1

	**Linear regression coefficients: EDSS**				
	**B**	**ES B**	**β**	**t**	**sig**
**TC**	-0,006	0,007	-0,123	-0,835	0,409
	R^2 = .211; F model = 2.541, p = .055				
**TG**	0,005	0,007	0,129	0,713	0,481
**LDL**	-0,017	0,012	-0,242	-1,444	0,158
**HDL**	0,007	0,015	0,07	0,462	0,647
	R^2 = .171; F model = 1.882, p = .112				

Model controlling for age, gender and BMI; SE B: standar error for B coefficient; TC: total cholesterol, TG: triglycerides, LDL: low density lipoprotein, HDL: high density lipoprotein
